# Fibroblastic osteosarcoma in a lion (*Panthera leo*)

**Published:** 2014-01-05

**Authors:** L. Leonardi, E. Lepri, S. Nannarone, O. Olivieri, L. Mechelli

**Affiliations:** 1 *Dipartimento di Scienze Biopatologiche e Igiene delle Produzioni Animali e Alimentari, Università degli Studi di Perugia, Italy*; 2 *Dipartimento di Patologia, Diagnostica e Clinica Veterinaria, Università degli Studi di Perugia, Italy*

**Keywords:** Bone tumor, Fibroblastic, Humerus, Osteosarcoma, *Panthera leo*

## Abstract

This report describes a case of spontaneous fibroblastic osteosarcoma in the humerus of a lion from a private park in Perugia, Italy. The tumor had an irregular, smooth, brown surface and a generally firm, rubbery consistence with gritty to hard areas interspersed. The mass was poorly vascularized with areas of necrosis at the periphery. The cut surface showed a multilobulated mass that had breached the humeral cortex, with periosteal production of reactive bone. The mass invaded the epiphysis, the synovial membrane, the joint capsule and ligaments. A mild hemorrhagic effusion appeared in the joint space. Clinical signs, gross and histopathologic findings are described in this rare case of a malignant bone tumor.

## Introduction

Osteosarcoma is the most common primary malignant bone tumor in animals and humans (Leonardi, 2003; Gilg *et al.*, 2013). Fibroblastic osteosarcoma is an aggressive tumor, which is frequently histologically indistinguishable from the conventional fibrosarcoma (Mehlman, 2012). Reports of neoplasms in *Panthera* species are still rare as neoplasms are uncommon causes of disease and death in captive wild felids. The presence of primary tumors in large felids is rarely reported, and there are few documented cases in the literature.

### Case details

A16 year old female lion (*Panthera leo*) in a zoological park became slightly lame in the right thoracic limb in March of 2011. The lioness was vaccinated against feline parvovirus, feline herpes virus type 1 and feline calicivirus. Clinical examination revealed a large, firm mass growing in the proximal third of the right humerus. The severity of the lameness increased over the course of several weeks and the animal was anesthetized with medetomidine (40 mg/kg) and ketamine (3 mg/kg), administered into the muscles of the hindquarters using a blowgun from a distance of about 10 meters. The animal became recumbent in about 10 minutes and was later intubated. Anesthesia was maintained with isoflurane in 100% oxygen.

**Fig. 1:**
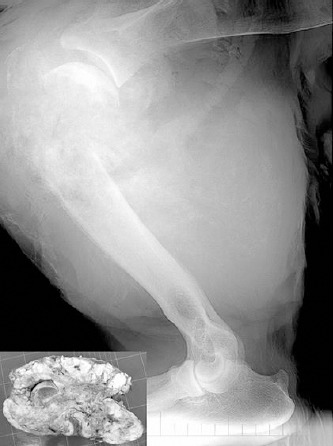
Mediolateral radiograph shows a destructive bone lesion with pathological fracture of the proximal third of the humerus. The tumor is densely sclerotic, containing a considerable amount of mineralized osteoid. Small picture in the inset shows gross appearance of the osteosarcoma, with destruction of the cortex and a soft tissue mass with osteogenic white areas.

A clinical examination of the leg revealed a severe swelling involving the shoulder, elbow and forearm area and a firm large mass growing in the proximal third of the right humerus. Radiographic examination of the right thoracic limb was performed with the lion in right lateral recumbency. Mediolateral views of the right shoulder, elbow, and humerus, and a lateral view of the thorax were obtained. The area over the proximal humerus was clipped and surgically prepared to obtain a fine needle aspiration (FNA) for the cytological evaluation of the mass. Radiographs of the limb showed an osteolytic sclerosing lesion with irregular rim, mild periosteal reaction and irregular cortex, intramedullary extension and pathologic fracture of the metaphyseal area, severe invasion and complete detachment of the right humeral epiphysis (Fig. 1).

**Fig. 2:**
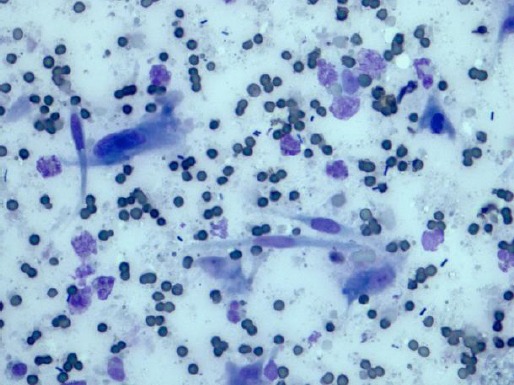
Fibroblastic osteosarcoma. Cytological specimen shows a moderately dense population of spindle cells isolated or arranged in small clusters with elongated scanty cytoplasm and plump oval nuclei. The nuclei contained evenly distributed chromatin and small nucleoli. Few osteoblasts and little production of bone matrix were detectable in the specimens examined (40x).

The radiographic examination of the thorax revealed no abnormalities. FNA from the mass yielded a moderately cellular sample composed of loosely cohesive groups or single spindle cells with basophilic cytoplasmic tails, oval nuclei with central prominent multiple nucleoli and mild anysokariosis (Fig. 2).

Based on these clinical, radiological and cytopathological findings the mass was diagnosed to be a primary malignant bone tumor, and the animal was euthanized and a complete necropsy performed. The tumor was 26 × 12 × 10 cm, with an irregular smooth, brown surface and a generally firm rubbery consistence with gritty to hard areas interspersed. The mass was poorly vascularized with peripheral necrotic areas. The cut surface showed a multilobulated mass that had breached the humeral cortex, with periosteal production of reactive bone. The mass invaded the epiphysis, the synovial membrane, the joint capsule and ligaments. A mild hemorrhagic effusion appeared in the joint space while the other organs were not affected. No metastases were detected during necropsy.

**Fig. 3:**
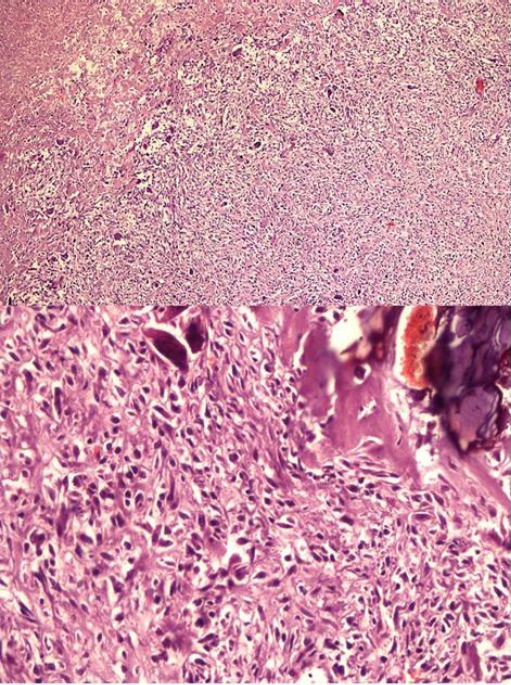
Fibroblastic osteosarcoma. The tumor shows a pattern of neoplastic mesenchymal cells producing osteoid, woven bone collagen with extensive osteoblastic differentiation. There are numerous fibroblastic cells associated with diffuse activation of osteoclastic reactive giant cells (H&E, 4x and 20x).

Histological examination showed malignant mesenchymal cells producing osteoid and woven bone. There was extensive osteoblastic differentiation associated with the production of collagen, as well as a population of fibroblastic-like cells associated with diffuse activation of osteoclastic giant cells. Neoplastic cells were large, pleomorphic and had hyperchromatic nuclei and prominent nucleoli. Where the tumor was more intensely osteogenic and sclerotic, the cells were rarer, and were small, with slender nuclei, dense chromatin and no mitoses (Fig. 3).

There was a diffuse and moderate infiltration of inflammatory cells including lymphocytes and monocytes, with fibroblasts arranged in a storiform pattern. Clinical and radiological findings including the growth rate of the lesion, and the histopathological appearance supported the diagnosis of fibroblastic osteosarcoma. This appears to be the first reported case of fibroblastic osteosarcoma in a lion.

According to WHO classification, osteosarcoma can be classified according to its site of origin in either the intramedullary, surface/juxtacortical or intracortical bone, either with epiphyseal, methapyseal or diaphyseal localization. Location may have a prognostic significance. In this case report it was not possible to identify the exact site of origin of the tumor. At the time of necropsy, it involved epiphyseal, methapyseal, and diaphyseal areas of the humerus and extended from the periostium and cortical bone to the medullary cavity.

## Discussion

The oncogenic mechanism of malignant bone tumors in domestic animals is controversial, and the biomolecular modifications involved in the development of the tumors are poorly understood. Rapid bone growth appears to predispose the animals to osteosarcoma, as suggested by the increased incidence during the adolescent growth spurt, the high incidence among large-breed dogs and osteosarcoma’s typical location in the metaphyseal area adjacent to the growth plate (physis) of long bones.

The only known environmental risk factor is exposure to radiation (Mehlman, 2012). There was no history of exposure to radiation in the animal investigated here, nor was there any indication of a genetic predisposition like bone dysplasias, fibrous dysplasia, enchondromatosis, multiple exostoses or mutations to RB gene or to p53. In domestic animals (Bitetto *et al.*, 1987; Helm and Morris, 2012), especially in dogs, the risk of osteosarcoma increases with age and with increasing body weight (Heldmann *et al.*, 2000; Leonardi, 2003; Leonardi *et al.*, 2012). The highest risk of osteosarcoma is found in large and giant breed dogs, and the incidence is lower in smaller breeds (Quigley and Leedale, 1983; Ru *et al.*, 1998).

Neoplasms of the bone are rare in wild felids, and the unusual subtype of the tumor makes this case even rarer. This case also confirms that the diagnosis of the specific subtype of osteosarcoma can only be reached by a multidisciplinary approach including radiology, pathology and clinical examinations.
